# The Next Era of Assessment Within Medical Education: Exploring Intersections of Context and Implementation

**DOI:** 10.5334/pme.1128

**Published:** 2024-10-09

**Authors:** Aliya Kassam, Ingrid de Vries, Sondra Zabar, Steven J. Durning, Eric Holmboe, Brian Hodges, Christy Boscardin, Adina Kalet

**Affiliations:** 1 Department of Community Health Sciences and Director of Scholarship in the Office of Postgraduate Medical Education at the Cumming School of Medicine, University of Calgary, Alberta, Canada; 2 Faculty of Education at Queen’s University, Kingston, Canada; 3 Division of General Internal Medicine and Clinical Innovation at the NYU Grossman School of Medicine, New York, New York, USA; 4 Center for Health Professions Education at the Uniformed Services University of the Health Sciences in Bethesda, Maryland, USA; 5 Intealth, Philadelphia, Pennsylvania, USA; 6 Temerty Faculty of Medicine at University of Toronto, Canada; 7 Royal College of Physicians and Surgeons of Canada, Canada; 8 Department of Medicine and Department of Anesthesia and Perioperative Care, and the Faculty Director of Assessment in the School of Medicine at the University of California, San Francisco, California, USA; 9 Department of Medicine, Center for the Advancement of Population Health at the Medical College of Wisconsin, Wisconsin, USA

## Abstract

In competency-based medical education (CBME), which is being embraced globally, the patient-learner-educator encounter occurs in a highly complex context which contributes to a wide range of assessment outcomes. Current and historical barriers to considering context in assessment include the existing post-positivist epistemological stance that values objectivity and validity evidence over the variability introduced by context. This is most evident in standardized testing. While always critical to medical education the impact of context on assessment is becoming more pronounced as many aspects of training diversify. This diversity includes an expanding interest beyond individual trainee competence to include the interdependency and collective nature of clinical competence and the growing awareness that medical education needs to be co-produced among a wider group of stakeholders. In this Eye Opener, we wish to consider: *1) How might we best account for the influence of context in the clinical competence assessment of individuals in medical education? and by doing so, 2) How could we usher in the next era of assessment that improves our ability to meet the dynamic needs of society and all its stakeholders?* The purpose of this Eye Opener is thus two-fold. First, we conceptualize - from a variety of viewpoints, how we might address context in assessment of competence at the level of the individual learner. Second, we present recommendations that address how to approach implementation of a more contextualized competence assessment.

## Introduction

In the most general sense, context constitutes the specifics of a given circumstance. This includes the individuals and the environment. The recent global pandemic has highlighted the important relationship between clinical competence and context. For example, in many instances highly experienced physicians were “redeployed” to care for patients with serious conditions outside their subspecialty domain of expertise [1,2,3] while at the same time very inexperienced clinicians, including senior learners, volunteered to graduate from medical school early to help care for the onslaught of very ill patients [4].

While the pandemic may be a singular and extreme example compared with what we faced on a daily basis before and since the global emergency, the many stories emerging from this experience underscore how clinical competence is interwoven with practice context; neither competence nor context are static; and thus, training a generation of doctors who can adapt their competence to different practice contexts is becoming ever more critical as new diseases emerge, new technologies are implemented, the negative health impact of climate crises accelerates and geopolitical conflicts threaten civilian populations.

In competency-based medical education (CBME), the context of the patient-learner-educator encounter can be considered at the micro- (individual), meso- (program) and macro- (system) levels [5]. Contextual factors moderate competence assessment and clinical outcomes in different ways at each level. Contextual factors may include the patient and their family members, other healthcare providers, other learners, multiple environmental inputs such as appointment length, electronic health records (EHRs), culture, and the interactions among these factors [6]. Recognizing and integrating contextual factors in real time allows us to explicitly embrace contextual diversity as part of assessment. It is important that context be considered in the moment assessment is undertaken since it is unlikely that we can later faithfully reconstruct the context in which decisions are made and from which actions follow hence [7].

Assessment of competence occurs in complex adaptive systems [8,9] that do not guarantee a particular result but rather predictably lead to a range of emergent outcomes that are highly interdependent with contextual factors. Of course, not all outcomes are equally desirable. Some variability, such as diagnostic error, is uniformly undesirable while other variability in outcomes may not be harmful, of equal benefit or even valuable and therefore acceptable. We concur with Van Melle and colleagues that holistic judgements based on a significant number of workplace-based assessments [10] be the dominant form of clinical competence assessment, as long as these judgements are made with care and attention to the many sources of performance and assessment variability including the vast array of contextual factors [10,11].

To account for context in competency assessment, it behooves us to solicit and incorporate the pluralistic viewpoints of key stakeholders throughout the process [12,13]. This process has been referred to as co-production [13,14,15], a vigorous and explicit commitment to *equitable integration of* input from a range of key stakeholders - including members of the profession, patients, learners, a range of health profession and systems partners in the design, implementation, and interpretation of competence assessment. This can be accomplished, for instance, by engaging key stakeholders in defining the standard data element categories for learner dashboards [16] including quantitative and qualitative data, educationally sensitive patient outcome measures, and learner sensitive quality measures [17,18,19] as well as determining policies including data governance, privacy and data use for clinical competence assessment.

Current and historical barriers to embedding context in assessment include the existing post-positivist epistemological stance in which we value measurement objectivity and validity evidence over context, most evident in the prevalence of standardized assessment of some competence areas such as medical expertise or communication at the exclusion of other relevant constructs. Bates and Ellaway point out that context has been elusive and subjective because it remains “largely invisible to those embedded in (it)” [20]. While always critical to medical education processes and outcomes, the centrality of context is especially pronounced as we have diversified training contexts and our conceptual view has grown beyond a simple focus on an individual’s competence to include consideration of the interdependencies and collective nature of competence [6,15].

In May 2022, a diverse group of scholars and institutional leaders in medical education and assessment, health informatics, and health services, gathered to identify innovation and research priorities for the next era of assessment in medical education [21] (Supplementary file 1: Appendix). As a group we initially identified four foundational themes including - *implementation and contextualization of assessment* the focus of this paper, as well as *accountability, trust, and power in assessment; harnessing the use of technology in assessment; and improving infrastructure for data sharing and open-source data*. Two days of intensive, thought-provoking discussions led to initial recommendations followed by a two-year process of refining our thinking through crafting manuscripts intended for peer review, and publication as part of this journal supplement [22]. The context and implementation working group, sought to explore: *How might we best account for the influence of context in the competence assessment of individuals in medical education?* and by doing so *How could we usher in the next era of assessment that improves on our ability to meet the dynamic needs of society and all its stakeholders?*

The purpose of this *Eye Opener* is thus two-fold. First, we conceptualize - from a variety of viewpoints on *how* to address context in assessment of competence at the level of the individual learner. Second, we present recommendations that address the role of context in the assessment of competence at the micro-level (individual learner) whilst considering the implications for the meso-level (programs) and macro-level (systems) implementation of competence assessment.

## Intersections of context with assessment of competence

While considering context can begin at any level, here we focus first on competence assessment at the level of the individual learner and then consider how this is impacted at the meso- and macro-levels. We propose that competence be defined as the elements of the interaction or interdependence of the learner and the learning environment that can be transferable to changing clinical care contexts [23,24,25].

In the next era of assessment, we must integrate the outcomes of healthcare and education [26]. We need to treat context as a central feature, rather than as a construct irrelevant variance or a potential confounder of our ability to measure competence. Within CBME, competence assessment that accounts for context can ensure that we are preparing future physicians to adapt to and thrive as clinicians in rapidly evolving complex healthcare delivery environments. We propose adding consideration of the following contextual factors into assessment, especially workplace-based assessment:

1. *Sociocultural elements* of the clinical encounter or learning environment itself including the dominant culture and language, location with respect to urbanity/rurality, and the socioeconomic status/features of individuals and the setting.
2. *Predictability* is associated with features of the environment (e.g. chaotic environments such as emergency rooms are predictably more distracting than clinical office environments) and the complexity of the problem. For example, (*a)* a simple problem has a known solution which when applied consistently leads to predictable outcomes (e.g. testing declarative knowledge with high quality standardized multiple-choice quizzes yields a usually predictable, proportion of learners needing remediation). Moreover (*b*) a complicated problem also has a predictable outcome but requires more effort (e.g. assessing technical/procedural expertise development *after* practice repetition, expert feedback and accumulated experience ensures high success and low complication rates). Last (*c*) a complex problem is one in which common solutions lead to unpredictable outcomes, therefore emergent problem solving is needed (e.g. assessing clinical reasoning in the face of a highly novel situation).
3. *Relational Autonomy* recognizes humans as interconnected and interdependent, and socially constructed. Whether or not an action is taken is dependent upon particular social relationships and the power structures in which the learner is embedded.
4. *Available equipment/technology* includes consideration of hardware as well as software (e.g. artificial intelligence). For example, whether there is a full suite of advanced diagnostic or procedural technology such as in an acute hospital, or only information technology such as in a community clinic or very limited access to all technology as in some geographical settings.
5. *Emotional tone* is the understanding of the emotional state and holistic wellbeing of the learner, patient and others in the learning and practice environment.

## Theories and Conceptual Frameworks that Support Context and Clinical Competence Assessment

In Table 1 and below we briefly discuss a set of selected theoretical and conceptual frameworks seriously considered in the health professions literature to provide useful lenses on how context interacts with competence assessment. However, first, we reiterate that in most cases co-production is the foundational process that will ensure the meaningful integration of context with, and successful implementation of, assessment.

**Table 1:** Theories and Conceptual Frameworks that able understanding of context and competence assessment with co-production.*


THEORETICAL FRAMEWORK	DESCRIPTION	HOW IT ACCOUNTS FOR CONTEXT IN ASSESSMENT

Situativity	Situativity theories include (1) situated cognition, (2) distributed cognition, (3) embodied cognition, and (4) ecological psychology. These distinct lenses can be applied simultaneously. Situated cognition is a theory that considers knowledge as constructed and interwoven with the context, to include the culture in which it is learned. Distributed cognition theory describes how knowledge is dispersed across the learning environment (e.g., with technology and other individuals), and all other factors external to the learner in the environment. Embodied cognition is a theory that attends to how sensory and motor inputs impact learning. Ecological psychology focuses on the learner-environment interaction and posits that cognition and learning emerge because of analysis of patterns used in a goal-directed task. Affordances in ecological psychology refer to how we perceive what environments have to offer and how these elements meet our needs.	Micro: Relational autonomy, emotional tone, predictability in assessment Meso: Predictability, available equipment/technology for assessment Macro: Sociocultural elements of assessment

Pattern Theory	Pattern Theory describes a learner’s cognitive processing, based on prior knowledge and how the learner accounts for variability and uncertainty in complex systems such as medical education. While the pattern demonstrated may be specific to a single learner, social systems in medicine and medical education create shared understandings and catalogues of patterns. Furthermore, sharing patterns of knowledge and learning and their accompanying attitudes and behaviours are part of the social system itself and thus relevant to contextualization theory.	Patient Patterns Learner Patterns Practice Patterns Institutional Patterns Educational Patterns Geographical Patterns Societal Patterns

**CONCEPTUAL FRAMEWORK**	**DESCRIPTION**	**HOW IT ACCOUNTS FOR CONTEXT IN ASSESSMENT**

Capabilities Approach	Human capabilities (as compared with competencies) broadly address how to enhance societal flourishing by maximizing what people can do and be. The capability approach is a theory that makes foundational claims: 1) The freedom to achieve health and wellbeing is of primary moral importance to the achievement of a good quality of life and, 2) Wellbeing should be understood in terms of people’s capabilities and functioning.	The Capabilities Approach helps understand practice variation among trainees with similar competence, how wellbeing impacts competence and defines contextual competence.

Workplace-based Assessment	The bulk of clinical competence assessment should take place in authentic environments (simulated or actual) where a high level of variability of assessments across raters and settings is meaningful and validity for the purpose of making judgements about competence of individuals is achieved with a high number of assessments.	Clinical competence assessments are made across a range of settings where most physicians practice and most healthcare services are delivered.

Complex Systems	Complex Adaptive Systems (CAS) are those in which stakeholders, policies, organizations, and other influencers, work together in non-linear and emergent manner continuously adapting and self-organizing.	Medical education is a complex adaptive system situated in relationship to a predictable set of other highly complex systems including, briefly, a higher educational system followed by a variety of healthcare systems. Relationships within and between systems and the dynamics of those relationships continually impact both the competence of individuals and teams and decision making about competence. Awareness of the interdependent, unpredictable, and emergent nature of CASs enhances contextualization of assessment.

Implementation Science	Effective implementation is best viewed as an integrated system, not the sum of its parts the Consolidated Framework for Implementation of Research (CFIR) identifies: Intervention characteristics Outer Setting Inner Setting Individual characteristics Implementation process	The CFIR and related determinant frameworks enable the identification of barriers and facilitators across multiple levels of context (learners, patients, providers, organization, and other stakeholder groups) facilitating the development of implementation strategies that increase the uptake of high-quality competency assessment.


*Co-production is a foundational process that facilitates contextualization in the next era of assessment.

### Situativity

Situativity is a family of social cognitive theories that conceptualize how contextual factors in the learning environment at the micro-level impact assessment [23,27]. Situativity theory addresses how learner experience interacts with the learning environment to impact knowledge acquisition, cognition, and other aspects of learning. Situativity acknowledges that both individual responsibility and participation in a community are essential to learning; the teaching and clinical environments are complex (i.e., non-linear, and multi-level); group interactions impact learning; and resources and tools such as data, medical equipment, technology etc., can and do impact learning.

### Pattern Theory

Bates and Ellaway propose six recurring contextual patterns in medical education that can guide the investigation and definition of context in medical education, [20]. These are: *1) Patient patterns* – the patients and clinical presentations encountered*, 2) Practice patterns* - the way clinical practice is most often structured as nested teams of individuals most proximally and immediately responsible for delivering care to patients *3) Educational patterns -* the different approaches to teaching and learning and their parent educational programmes and institutions*, 4) Institutional patterns* - the structures, financing and processes of healthcare institutions, 5) S*ocietal patterns* - the community’s organization, cultures, technologies, and values *and 6) Geographical patterns* - the physical positioning of training locations. Considering these six patterns as frames for the systematic investigation needed in the next era of assessment allows us to identify and characterize important nuances that impact the transfer of clinical skills across contextual factors (nested levels) that influence the competence of individuals.

### Contextual Competence and the Capability Approach

The relationship between the learner’s capabilities and the tasks they are required to perform in clinical settings must be assessed as “an activity that takes place in the moment and emergent from an interplay of individual and contextual factors, including the community to be served” [24]. From this perspective contextual competence, or the ability to transfer competence from one setting to another, results from the learner’s ability to attend to their own holistic wellbeing [28] and practical needs, followed by a sense of belonging and interdependence, and “finally a re-constitution of competence” in the new context [24]. A trainee’s capacity to regularly maintain or regain clinical competence in response to new circumstances across their careers can be assessed [15,16]. This capacity provides a conceptual connection between technical competence, capability, and wellbeing across locations of practice [15,17].

The capability approach —a theoretical framework first defined in economics and applied to public health ethics focuses on what people can do and be, helps us understand contextual competence. Theoreticians Nussbaum and Sen define capability as “the combination of individuals’ ability and the opportunities and constraints they encounter in using their abilities” [29]. This approach shifts the focus from simply having the means (e.g. training, wellbeing) or competence (determined only in one context) to be competent toward ensuring that both the material (e.g. resources, training, tools) and non-material (e.g. rights, permissions, freedom from legal consequences) context enables the full expression of that ability. Personal and contextual factors help to explain why two learners with similar training (e.g. curriculum, rotations) may not achieve the same educational outcomes. This also implies how to think holistically about the interaction between wellbeing and clinical competence in medical education and healthcare [8,28,30,31].

### Workplace Based Assessments

Except for the earliest stage of medical education, physicians develop their clinical competence in the authentic, clinical environments where healthcare is delivered. Therefore, it follows that this is where most of the clinical competence assessment could take place. For the past two decades, Workplace Based Assessments (WBA) have been widely implemented in the postgraduate medical education setting but there is still great variation in implementation and significant gaps in our knowledge of best WBA practices [32] especially regarding how to take context into account. In the next era of assessment, we should focus on increasing the contextualization, quality, quantity, and impact of both direct and indirect WBA [31,33].

### Complex Systems

Medical education is a Complex Adaptive System (CAS) [8,9,34] interdependent with other extremely complex healthcare systems. During the recent pandemic, for example, the interactions between learners and teachers in both the healthcare systems and medical education system were perturbed, not only by their individual roles and personal attitudes, but also by educational and public health policies, patient involvement, political challenges, and pandemic-related advances in management. It is system characteristics - non-linearity, interactions, and interdependencies- that prevent us from fully predicting the future in a CAS [25]. However, accepting and seeking to understand the complexity is a path toward embracing context in assessment.

### Implementation Science

Implementation science, defined as the “study of methods to promote the systematic uptake of research findings and other evidence-based practices into routine practice…” [35], provides a framework for designing clinical competence assessment systems that are fully embedded in the healthcare workplace context.

**Figure 1:**
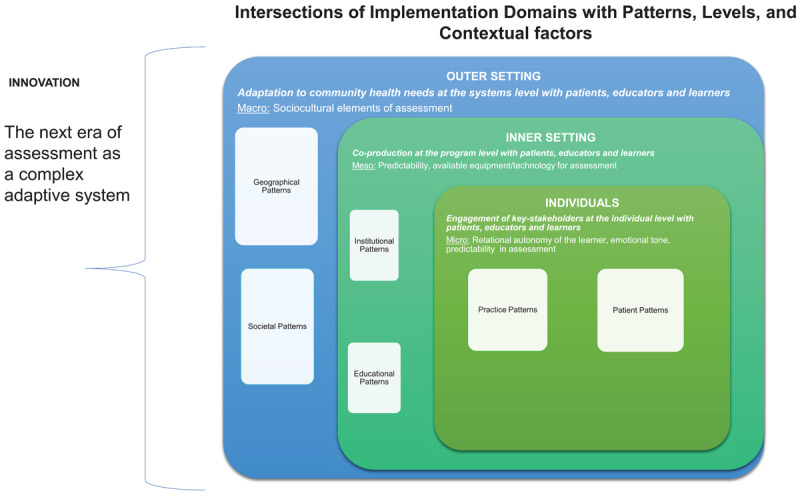
Intersections of Implementation Domains with Patterns, Levels, and Contextual factors.

When designing and implementing the next era of assessment of medical education, we recommend the Consolidated Framework for Implementation of Research (CFIR) [36,37] be used for the duration of the implementation, from planning, preparation, implementation, and during post-implementation evaluation. Figure 1 depicts the intersections of the CFIR implementation domains with patterns, micro-, meso- and macro-levels, and contextual factors.

During the preparation phase of the new assessment, CFIR directs the identification of barriers and facilitators in the service of guiding selection of appropriate mitigation strategies. Throughout implementation, CFIR guides data collection (e.g., surveys, interviews, and observations) and analyses, providing stakeholders with feedback. CFIR, originally developed in 2009, and revised in 2022 [35], defines the key domains that should be addressed when determining barriers and facilitators at multiple levels that may impact implementation outcomes [36,37]. Figure 1 shows how CFIR domains can help clarify patterns, levels, and contextual factors by considering the following:

1. *Innovation domain*: a detailed description of the innovation, e.g., incorporating contextual variation into assessment strategies. Consider: Why is this assessment better? Does it require new concepts or technology?
2. *Outer setting domain*: (see macro-level in Figure 1), a detailed description of the setting at the systems levels e.g., new policies that must be developed. Consider: What societal pressures are driving this change?
3. *Inner setting domain*: (meso-level described in Figure 1), a detailed description of the specific setting(s) where the innovation is to be implemented, e.g., in clinic or simulations. Consider: What are the concerns about the new assessment? What resources are needed and available to support the change?
4. *Individuals domain* (see micro-level in Figure 1): a detailed description of the roles, beliefs and values of the individuals involved in the innovation, e.g., learners, patients, healthcare providers, and other individuals who have influence over the implementation outcomes in the next era of assessment. Consider: Who will be using the new assessment? What are their attitudes towards the innovation?
5. *Implementation process domain*: a detailed description of implementation strategies and tactics. Consider: What are the key steps to roll out the new assessment? How will feedback be gathered?

## Discussion

The next era of assessment should expand the number of contextual factors considered to best serve patients and learners. For instance, we suggest including sociocultural elements, predictability, relational autonomy, availability of equipment and technology, and emotional tone, among others. But the best ways to accomplish this goal are not yet clear. In this Eye Opener we describe how we might account for the influence of context in the competence assessment of individuals in medical education. First, we establish the connection between selected theories and conceptual frameworks and assessment. We suggest that contextualized assessment should take place in workplace-based settings and describe how to embrace and navigate contextualized assessment in a complex adaptive system focusing on the level of the individual learner. We argue that the co-production process and an implementation science framework are foundational elements to implementing contextualized clinical competence assessment to better serve patients and learners.

In a holistic sense, contextualization occurs as part of the complex adaptive system, yet specific instances of assessment do occur at each of the micro-, meso- and macro- levels. For example, contextual factors can be seen as occurring as part *of* assessment, *for* assessment and *in* assessment whilst considering the patterns within and across the broader context using the CFIR framework.

For example, an experienced learner such as a senior resident physician, new to a clinic setting and unfamiliar with its EHR and the cultural values and dominant language of the community, may become less able to provide competent care for patients with routine medical issues. These contextual factors may impact the patient’s outcome. Learners are likely to vary in how well they navigate and adapt to unpredictable and predictable changes in the care environment. Given the emergent nature of complex adaptive systems, assessments should include not only predictable constructs and settings, but also those that are unpredictable (e.g., unexpected patient behaviour) or unfamiliar (e.g., a new version of the electronic health record). The common, current approach when a patient displays an unexpected behavior is to adjust the assessment. By actively incorporating the unexpected into the assessment, we can help the trainee learn how to deal with such situations.

This *Eye Opene*r is not without limitations. First, the diverse group of scholars in medical education that were present during these discussions while “experts”, did not include any learners or represent patients. Key stakeholder engagement in the next era of assessment cannot be over emphasized. Second, while we provide recommendations that are scaffolded upon theoretical and conceptual frameworks, they are just that – theories and concepts and thus we cannot speak to their feasibility in real-world contexts where the entrenched structures and culture of medicine still prevails. Despite this, we hope we have provided insights that will help medical educators realize the importance of context, co-production and implementation science in the next era of assessment.

We encourage further critique and discourse around our blue sky thinking and the notion that competence assessment occurs in a complex adaptive system where relationships are not linear. We acknowledge the discomfort that may arise in our circular conclusion that *context and assessment should drive each other*. We urge readers to consider their reactions to our claim that assessment cannot be valid unless context is considered. Ideally, in the next era of assessment a criterion for establishing assessment validity will be public and professional accountability.

## Contextualizing Assessment: Recommendations

Contextualizing assessment requires considering everything that can impact the learning environment or clinical encounter between the learner and the patient. If context is everything and everything is context, where do we begin? Practically we should start where we are and focus on our current learners and trainees, the near future healthcare workforce, to prepare them to practice with contextual competence.

### Recommendations for Incorporating Context Through Co-Production of Assessment

While we have argued that co-production is a fundamental process in the next era of assessment the implementation of any changes in assessment usually is the responsibility of educational leaders, who must navigate resource limitations including time, money and people/expertise and have limited longitudinal relationships with learners. The following recommendations of our group represent initial, pragmatic steps to help navigate the many tensions that will inevitably arise:

1. Convene a diverse set of stakeholders, with meaningful involvement of patients and learners [12], in a learning community committed to building an audacious, high value, longitudinal and adequately resourced and fully contextualized next era assessment system.
2. Adapt and expand metrics on clinical outcomes, patient experiences and related work to put in the hands of medical educators for use in substantive feedback conversations with learners about blind spots, resilience and contextual competence [38].
3. Seek evidence for contextual competence in both data collected intentionally as assessment and data not initially intended for assessment such as patient experience and other Electronic Medical Record (EHR) data.
4. Ensure some assessment data is derived from standardized simulated scenarios that allows educators to study contextual competence.
5. Study the relative influence of micro-, meso- and macro-levels of context during a clinical encounter in a workplace-based setting for both formative and summative assessment.
6. Intentionally reflect on how contextual factors may meaningfully contribute to clinical care outcomes and incorporate these contextual factors into assessment [26].

Fully embracing contextual factors and patterns in the next era of assessment in medical education, will be as Hall et al. (2020) reminds us “…a marathon, not a sprint…” [39]. Many challenges will emerge as we implement new assessment policies and strategies. As has been our experience in the introduction of CBME, challenges are seen at many levels. [36,40]. For example, despite their theoretical value, learners, resident physicians and faculty members, struggle with the administrative burden of completing frequent low-stakes assessments [41]. Faculty, program directors and learners have found it difficult to fully change their outlooks from a fixed mindset to a growth mindset needed to implement CBME [42]. We believe that with a rigorous implementation framework in place, time and intellectual flexibility, contextualizing assessment is likely to be fruitful.

## Conclusion

Ideally in the next era, all key-stakeholders will be engaged in the co-production of assessments that are highly contextualized and center on how individuals and groups of learners consistently demonstrate competence that is adaptable across contexts and in the face of real-world challenges and complexity. These innovation and improvement efforts should be continuous and driven by societal needs.

## Additional File

The additional file for this article can be found as follows:

10.5334/pme.1128.s1 Supplementary File 1. Appendix.
