# Supplementary material for: The Next Era of Assessment Within Medical Education: Exploring Intersections of Context and Implementation
Source: Perspect Med Educ. 2024 Oct 9;13(1):496–506. doi: 10.5334/pme.1128 (PMC11469546; doi:10.5334/pme.1128)
Supplement: Supplementary File 1. — Appendix. [file pme-13-1-1128-s1.pdf]

Next Era in Assessment

TECHNICAL REPORT ON MAY 23-25, 2022 MEETING

In May 2022, the Kern Institute for the Transformation of Medical Education at the Medical College of Wisconsin and the NBME convened a meeting of leaders of regulatory bodies, national associations and researchers engaged in advancing assessment in medical education. The meeting took place from May 23 to 25, 2022, in Milwaukee, Wis., with several participants joining remotely. The aim of this meeting was to help define the next era of assessment in medical education. The impetus for this gathering was a two-day virtual conference in April of 2021 that explored the role of electronic health record data in revolutionizing medical education assessment practices by placing the patient at the center of assessment. The group determined that a coordinated approach was required to address this challenge and opportunity.

Cambridge-style meeting

We designed this meeting to create an atmosphere of collaboration and maximize the participants' freedom to define the agenda and the nature of the discussions thereby unleashing creativity and innovative thinking. Modeled on the Cambridge Conference, a meeting style started in 1928 hosted by the Ordnance Survey, we sought to bring senior leaders of invested parties together to envision the future of assessment in medical education.

The Josiah Macy Jr. Foundation, which regularly sponsors similarly styled conferences, provided advice and planning expertise. The agenda (Fig. 1) was loosely structured to ensure sufficient time for large group brainstorming and brief topical presentations, small group discussions and social activities. In preparation for the meeting, the advisory group provided a document describing the common challenges faced by the medical education assessment community and along with a vocabulary for the meeting. In addition, each attendee wrote a vision statement for the next era in medical education assessment and provided brief personal biographies, which were distributed to all attendees prior to the meeting.

On the first day of the meeting the participants as a group identified the most pressing issues in assessment. Each participant voted for the issues that they perceived to be the most important. The themes that received the most votes were used to form four small groups: (1) contextualization and implementation of assessment, (2) accountability, trust and power in assessment, (3) data visualization, portfolio, data sharing, open-source data, data infrastructure, and (4) the use of technology in assessment and burden on assessors. In addition to the four major themes, participants identified several cross-cutting topics that were relevant to all discussions around assessment, including (1) diversity, equity and inclusion and bias mitigation, (2) value to the patient, (3) wellness and accountability to the patient and learner, (3) the impact on/of selection.

On subsequent days, participants worked in small groups to further elaborate on each of the four themes while incorporating the cross-cutting themes in their discussions to identify practical recommendations to advance assessment in medical education. This report summarizes these discussions below.

Key recommendations

i. The role of context in implementing the next era of assessment

Training a generation of doctors who can adapt their competence to different contexts is becoming ever more critical as events like the global pandemic arise, new technologies are implemented, new diseases emerge and the health impact of climate crises accelerates. We need to bring context back into assessment, rather than ignoring it or actively trying to standardize it, so that assessment in medical education can embrace the context-rich dynamic environments that are emphasized in competence-based models of medical education. Standardized assessments should have a fit for purpose role in an overall program of assessment. By increasingly emphasizing context as a central feature in assessment, we are preparing future physicians to adapt to complex health care environments.

Health care and medical education systems by nature of their non-linear, unpredictable properties, can be considered as Complex Adaptive Systems (CAS). Patients, health care team members and faculty continuously interact with each other individually and as teams; those interactions have future implications that are not always considered in assessment.

We propose creating a feasible and useful implementation plan to center the next era of assessment on patients and learners in internal and external contexts.

We propose creating a feasible and useful implementation plan to center the next era of assessment on patients and learners in internal and external contexts. To achieve this, all those invested in the success of medical education and its assessment should provide input, including, but not limited to, patients, learners, health care professionals, electronic health record (EHR) vendors, health systems leaders, educators, and accreditors.

Our recommendations for implementation of context into assessment include:

(1) Add, specify, or make note of context in all aspects of assessment, including prospective/ repurposed data and EHR data. Identify the relative power of contextual factors, e.g., location, time/day, fatigue, distractions, race/ethnicity/gender, mental health indices; these factors can be introduced progressively into simulation scenarios until productive failure is achieved. Balance the focus towards the ambulatory setting where most physicians practice and most health care is delivered.

(2) Co-produce assessment with patients and learners, integrating education and patient outcomes. Engage patients in a meaningful manner in their role of training health care professionals. Tools derived from patient experiences, desires, values, behaviors (e.g., Patient Reported Outcomes Measures, PROMs)) should be used to help teachers provide meaningful feedback to learners.

(3) Identify key stakeholders and other resources that could be available to build the next era assessment, and who will defend the value and affordability of this effort. The proposed data sources should be fit for purpose, have appropriate validity evidence and be supported by EHR vendors. Successful implementation, maintenance and continuous improvement of next era systems will rely on defined policies, including data governance, privacy and use.

When designing and implementing the next era of assessment of medical education, we recommend using the Consolidated Framework for Implementation Research (CFIR) to identify barriers and facilitators in the preparation phase, guide data collection and analyses throughout implementation, and as a framework for outcomes evaluation in the post-implementation phase.

ii. Accountability, trust and power in assessment

This theme centers itself around the lack of trust that has been created by the previous eras of assessment, current assessment practices across the medical education continuum, as well as the lack of transparency at all levels of assessment.

Main contributors to the lack of trust in medical education assessment include:

(1) institutional conflicts of interest,
(2) lack of learner ownership in the process,
(3) stakes associated with assessment/competitive selection,
(4) lack of relevance of assessment data,
(5) lack of transparency,
(6) lack of educational adjustments to assessment evidence,
(7) lack of a competency continuum framework,
(8) and insufficient data resources and literacy.

We propose that trust can be rebuilt by thinking of assessment as a system as well as defining an overall clear purpose, the elements that contribute to a trustworthy system and how these elements are all interconnected. Assessment needs to be aligned with values focused on the benefit of the patient at all levels of training. By protecting the integrity of the intended use and purpose for assessment and communicating often between stakeholders, we can achieve greater transparency and accountability in assessment.

The levels of the system where the trust needs to be (re)built include:

(1) micro/personal (i.e. learner, teacher, patient);
(2) meso/social (i.e. teams, course/rotation, local community);
(3) macro/organizational (i.e. program or institution);
(4) global (i.e. health systems, regulators, society)

Changes on the macro level are deemed to have the biggest return on investment as they would have cascading effects on the other levels. Therefore, practical recommendations include targeting changes on this level. Any implementation of changes at the macro level, however, needs to be considered next to the administrative and financial burden that this places on the individual programs and program directors.

Recommendations for rebuilding trust and accountability in medical education assessment include:

(1) Applying principles of designing trustworthy organizations to assessment in medical education can help create a trustworthy assessment system for a program or an institution.
(2) Improving trust, power and accountability on the program/institution level needs to include development of the faculty, the learners and the program.
(3) Faculty development should focus on placing coaches in the center of trustworthy assessment in medical education. Coaches would ensure that data provided to learners is not just a data point but rather a conversation starter to stimulate reflection and growth.
(4) Learner development is needed for learners to build trust, take ownership and actively participate in the process of assessment. Learner development should focus on assessment literacy for the learners.

By protecting the integrity of the intended use and purpose for assessment and communicating often between stakeholders, we can achieve greater transparency and accountability in assessment.

Particularly, learners should understand the purpose of the assessment, how it affects the learner and how it affects the patients the learner is caring for.

(5) Program development is needed to ensure there is consistency and coherence across assessments within a medical school as well as across all medical schools, programs and systems, which would build trust in learners regardless of the learner location and as they move through training from undergraduate medical education to graduate medical education.

iii. Data visualization, portfolio, data sharing, open-source data, data infrastructure

We need data to make decisions about learners. For example, in the form of a portfolio that the learner takes with them throughout their training, regardless of where they go. Educational and clinical data in medical education is used not only for student learning, but also for faculty development, promotion decision making, program evaluation and research/scholarship.

In addition to the practical uses of data sharing in medical education, there is also a moral argument for doing so. If learners are considered "owners" of their data, it is the responsibility of the programs to provide them with valuable feedback/data over time that learners can leverage later in their career. For this reason, data the learners hold should be compiled and presented in a useful way while being transferrable from between training sites and stages of training. Moreover, there are institutional benefits of sharing data on learners that include being able to do research using pooled datasets, leading to less chance for bias and more generalizable conclusions about learners' performance, etc.

In addition to the practical uses of data sharing in medical education, there is also a moral argument for doing so. If learners are considered "owners" of their data, it is the responsibility of the programs to provide them with valuable feedback/data over time that learners can leverage later in their career.

Often, fears of data breach get in the way of data being useful for learner, patient populations and institutions resulting in "silos" separating clinical from educational data. Furthermore, the lack of data sharing between institutions and across undergraduate, postgraduate and continuing training environments thwart any efforts to improve the use of educational and clinical data for these purposes.

Recommendations on the use of data for medical education assessment include:

(1) Centralization of data to help overcome practical challenges associated with the application of new techniques of machine learning and artificial intelligence. While some successful cases of data sharing exist, many of these efforts to broadly share data across institutions are limited by challenges including funding, data governance, protections from unintended uses, data standards, technical resources, and consent.

(2) Assessment data and clinical data should be collected within an organized architecture, shared in a secure and ethical fashion, linked to relevant external data sources, and organized to support its usage for the development of trainees, faculty, programs, specialties and institutions while improving patient care. Even if we wanted to share the data over time, it is a huge task to store the data. There are issues regarding formatting, how things are linked up, differences in security, etc. If we could overcome these challenges, we could pool our data.

(3) A proposed framework for data sharing should be based on generalizable principles. Implementations may vary, but guiding rules and principles should remain for anyone or any entity storing or sharing data for any purpose in medical education.

(4) An action-oriented blueprint can overcome challenges to organizing and visualizing educational and clinical data. Examples of success overcoming these challenges can be contextualized using case studies as well as data maturity frameworks and models from disparate industries. This blueprint can be used as a road map for others developing educational and clinical data infrastructure to support individual and organizational development.

iv. The use of technology in assessment

Technology holds many promises in assessment, yet it means many different things to different people. In programmatic assessment, technology can be used to gather more evidence of a learner's competency. In addition, technology can be used to expedite the provision of meaningful feedback to learners. For high stakes assessment, organizations like the NBME in the United States regularly use technology to improve various assessment tasks (link).

There is a temptation to use technology for many purposes — a hammer seeking a nail. The challenge of applying new technologies in assessment is addressing actual problems related to collecting, analyzing and reporting on data and the consequences associated with using an assessment.

Recommendations on the use of technology in medical education assessment include:

(1) Use of technology in assessment offers:
   a. the opportunity for scalability;
   b. the reduction of menial human-performed tasks that allows each user of the technology to reduce burden and practice at the top of his/her license;
   c. a new source of data with the result of increased objectivity;
   d. improvement in feedback quality and quantity;
   e. mitigation of bias in assessment;
   f. the reduction of assessor burden by providing new way to aggregate and report data (e.g. administrative replacement, data analysis, decision-making support for promotion/advancement in training, quality control and reduction of burden on assessors).

(2) Clarify the ultimate purpose for integrating technology into assessment: certification, support the improvement in learning and performance in each individual resident, with the additional caveat that it be done so in the service of the patient.

(3) In the service of learners, assessors and patients, technology should:
   a. be credible, valid and trustworthy;
   b. lead to measurably improved outcomes and do so in a manner that is usable;
   c. be workflow neutral if not workflow positive.

(4) In harnessing technology for assessment, it is important to consider the ownership, maintenance and definition of bias within the data.

(5) Additional affordances created by technology need to consider the costs of developing or acquiring additional software, training and learning curves.

(6) Assessment developers and implementers need to be guided in understanding the core elements of using a technology-based assessment in the context of medical education.

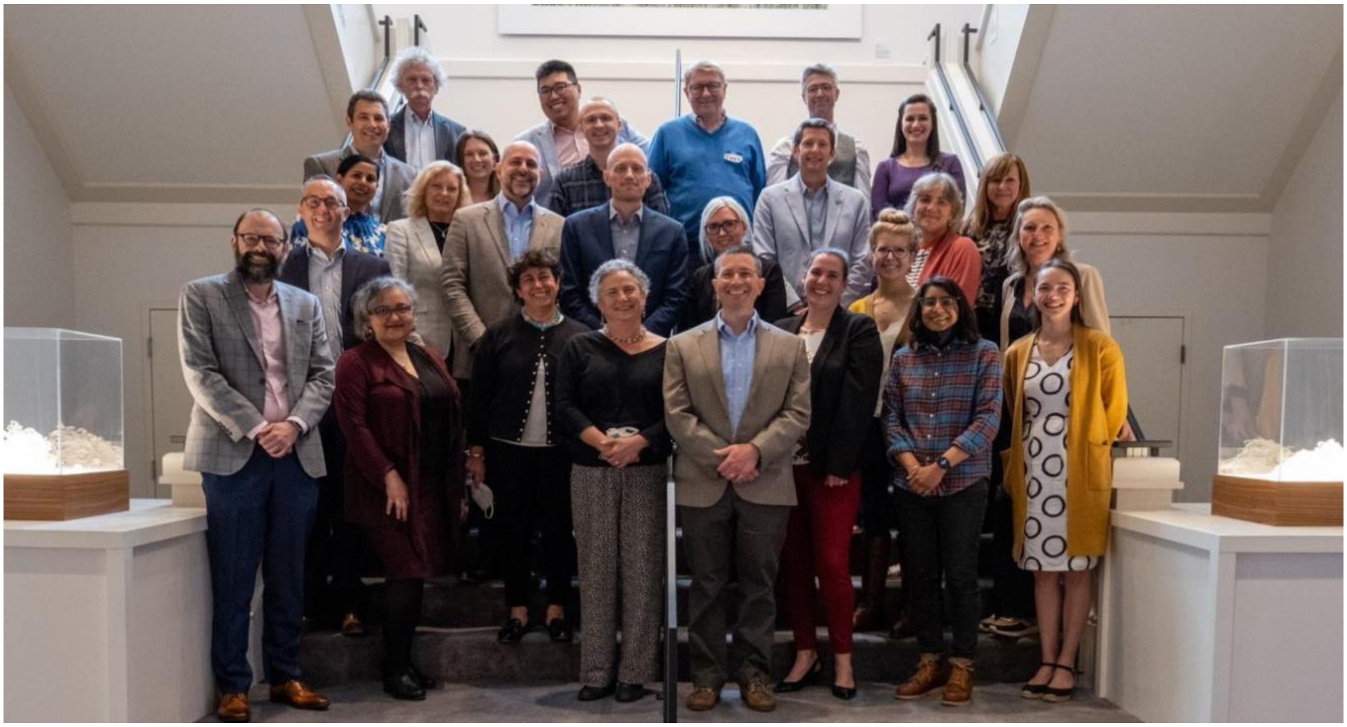

Attendees

Dr. John Andrews
Vice President for Graduate Medical Education Innovations, American Medical Association

Dr. Tavinder Ark
Assistant Professor; Director of the Medical Education Data Science Lab, Robert D. And Patricia E. Kern Institute for the Transformation of Medical Education, Medical College of Wisconsin

Ms. Cassandra Barber
PhD candidate, School for Health Professions Education, Maastricht University

Dr. Michael Barone
Vice President for Competency-Based Assessment, NBME

Dr. Christy Boscardin
Director of Student Assessment in the School of Medicine, Professor, Department of Medicine and Anesthesia, Office of Medical Education, University of California San Francisco, deputy editor for Teaching and Learning in Medicine, and statistical editor for Perspectives on Medical Education

Dr. Holly Caretta-Weyer
Director of Evaluation and Assessment, CBME Implementation Lead, Associate Residency Program Director for the Stanford University Emergency Medicine Residency Program, for the Stanford University School of Medicine, and PhD candidate, School for Health Professions Education, Maastricht University

Dr. Saad Chahine
Associate Professor of Measurement and Assessment at the Faculty of Education, Queen's University

Dr. Brian Clauser
Distinguished Research Scientist in the Office of Research Strategy, NBME, fellow of the American Educational Research Association and past editor of both the Journal of Educational Measurement and the NCME book series

Dr. Ethan Duwell
Postdoctoral fellow, Medical Education Data Science Lab, Robert D. And Patricia E. Kern Institute for the Transformation of Medical Education, Medical College of Wisconsin

Dr. Chris Feddock
Associate Vice President for Competency-Based Assessment, NBME

Dr. Jason Frank
Director, Centre for Innovation in Medical Education, University of Ottawa, and Past Director, Specialty Education, Strategy and Standards in the Office of Specialty Education, Royal College of Physicians and Surgeons of Canada

Dr. Brian George
Assistant Professor of Surgery, Chief of Trauma, and the Director of the Center for Surgical Training and Research, University of Michigan, and Executive Director, Society for Improving Medical Professional Learning

Dr. Polina Harik
Senior Measurement Scientist, NBME

Dr. Tina Hernandez-Boussard
Associate Professor at Stanford University in Medicine (Biomedical Informatics), Biomedical Data Sciences, Surgery and Epidemiology & Population Health (by courtesy)

Dr. Brian Hodges
Executive-Vice President Education and Chief Medical Officer, University Health Network, and Professor, Faculty of Medicine, University of Toronto

Dr. Eric Holmboe
Chief, Research, Milestones Development and Evaluation Officer at the Accreditation Council for Graduate Medical Education, Adjunct Professor of Medicine, Yale University School of Medicine and the Uniformed Services University of the Health Sciences, and Adjunct Professor of Medical Education, Feinberg School of Medicine, Northwestern University

Dr. Adina Kalet
Stephen and Shelagh Roell Endowed Chair and Director of the Robert D. And Patricia E. Kern Institute for the Transformation of Medical Education, Medical College of Wisconsin

Dr. Aliya Kassam
Associate Professor and Research Lead, Office of Postgraduate Medical Education, Cumming School of Medicine, University of Calgary and Equity, Diversity, and Inclusion Lead, O'Brien Institute for Public Health, University of Calgary

Dr. Andrew Krumm
Assistant Professor of Learning Health Sciences, Assistant Professor of Information, and Affiliated Faculty with the Michigan Institute for Data Science, University of Michigan

Dr. Hollis Lai
Associate Professor, Director of Learning Sciences, MD Program, and Director of Innovation and Quality Improvement, School of Dentistry, University of Alberta, Vice Chair, Committee on Learning Environment, University of Alberta, and Co-Chair, Data Stewardship Committee, Association of Faculties of Medicine of Canada

Ms. Dana Levinson
Chief Program Officer, Josiah Macy Jr. Foundation, and past Associate Dean, Medical School Administration, University of Chicago Pritzker School of Medicine

Dr. Kiki Lombarts
Professor Physicians' Professional Performance, Chair of the Professional Performance & Compassionate Care research group, Department of Medical Psychology, University of Amsterdam, Amsterdam University Medical Centers, 2020-21 Center for Advanced Study in the Behavioral Sciences and Fellow at the Presence Center, Stanford University

Dr. Kimberly Lomis
Vice President for Undergraduate Medical Education Innovations, American Medical Association

Ms. Abigail Martini
Clinical Research Coordinator, Cincinnati Children's Hospital Medical Center

Dr. Benjamin Rosner
Associate Professor of Medicine, informaticist and digital health researcher, Center for Clinical Informatics and Improvement Research, University of California San Francisco

Dr. Jonathan Rubright
Vice President of the Office of Research Strategy, NBME

Dr. Abigail Schuh
Associate Program Director and Assistant Professor of Pediatrics, Medical College of Wisconsin

Dr. Dan Schumacher
Associate professor, Division of Emergency Medicine, Cincinnati Children's Hospital Medical Center, and Co-Director of the Education Research Unit and Education Research Scholars Program, Cincinnati Children's Hospital Medical Center

Dr. Stefanie Sebok-Syer
Assistant Professor, Department of Emergency Medicine, Stanford University

Dr. Alina Smirnova
Clinical Assistant Professor, Department of Family Medicine, University of Calgary, and Adjunct Assistant Professor, Robert D. And Patricia E. Kern Institute for the Transformation of Medical Education, Medical College of Wisconsin

Dr. Brent Thoma
Professor of Emergency Medicine, Department of Emergency Medicine, University of Saskatchewan, and Clinician Educator, Royal College of Physicians and Surgeons of Canada

Dr. Marc Triola
Associate Dean for Educational Informatics and Associate Professor of Medicine, NYU Grossman School of Medicine, and Founding Director of the Institute for Innovations in Medical Education

Dr. David Turner
Vice President for Competency-Based Medical Education, American Board of Pediatrics, and Consulting Professor, Department of Pediatrics and Division of Pediatric Critical Care, Duke Children's Hospital

Dr. Cees van der Vleuten
Professor of Education and past Chair, Department of Educational Development and Research, Faculty of Health, Medicine and Life Sciences, Maastricht University and past Scientific Director of the School of Health Professions Education, Maastricht University

Ms. Ingrid de Vries
PhD candidate, Faculty of Education, Queen's University

Dr. Viktoriya Yaneva
Senior Data Scientist, NBME and Honorary Research Fellow, University of Wolverhampton

Dr. Sondra Zabar
Professor of Medicine, Director of the Division of General Internal Medicine and Clinical Innovation at NYU Grossman School of Medicine

Fig. 1 Agenda

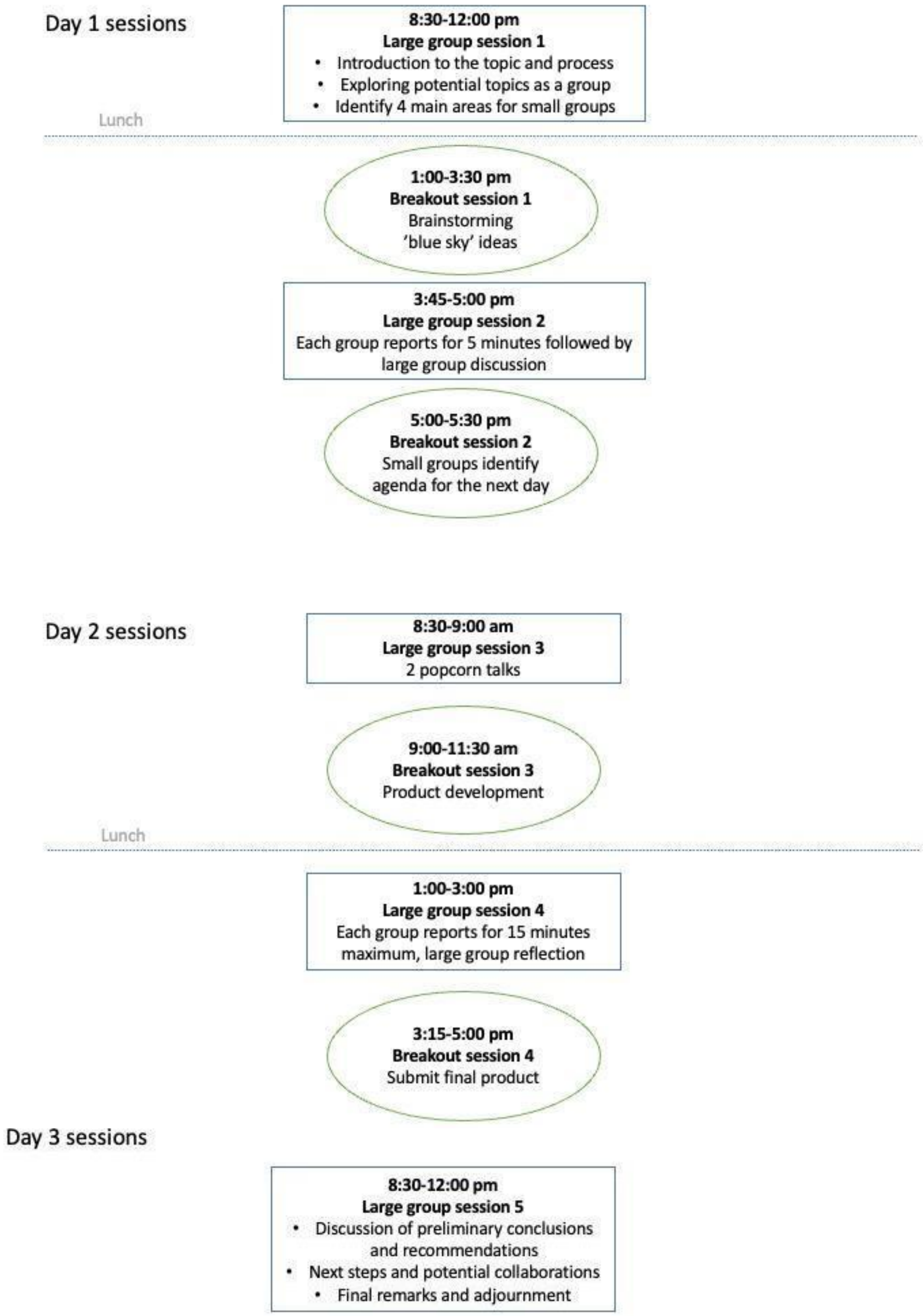

Day 1 sessions
8:30-12:00 pm
Large group session 1
• Introduction to the topic and process
• Exploring potential topics as a group
• Identify 4 main areas for small groups
Lunch
1:00-3:30 pm
Breakout session 1
Brainstorming
'blue sky' ideas
3:45-5:00 pm
Large group session 2
Each group reports for 5 minutes followed by large group discussion
5:00-5:30 pm
Breakout session 2
Small groups identify agenda for the next day
Day 2 sessions
8:30-9:00 am
Large group session 3
2 popcorn talks
9:00-11:30 am
Breakout session 3
Product development
Lunch
1:00-3:00 pm
Large group session 4
Each group reports for 15 minutes maximum, large group reflection
3:15-5:00 pm
Breakout session 4
Submit final product
Day 3 sessions
8:30-12:00 pm
Large group session 5
• Discussion of preliminary conclusions and recommendations
• Next steps and potential collaborations
• Final remarks and adjournment
